# Association of Chemotherapy, Enzalutamide, Abiraterone, and Radium 223 With Cognitive Function in Older Men With Metastatic Castration-Resistant Prostate Cancer

**DOI:** 10.1001/jamanetworkopen.2021.14694

**Published:** 2021-07-02

**Authors:** Shabbir M. H. Alibhai, Henriette Breunis, Gregory Feng, Narhari Timilshina, Aaron Hansen, Padraig Warde, Richard Gregg, Anthony Joshua, Neil Fleshner, George Tomlinson, Urban Emmenegger

**Affiliations:** 1 Department of Medicine, University Health Network, Toronto, Ontario, Canada; 2 Radiation Medicine Program, University Health Network, Toronto, Ontario, Canada; 3 Department of Medical Oncology, Kingston Regional Cancer Centre, Kingston, Ontario, Canada; 4 Kinghorn Cancer Centre, Darlinghurst, New South Wales, Australia; 5 Department of Surgical Oncology, University Health Network, Toronto, Ontario, Canada; 6 Biostatistics Research Unit, University Health Network, Toronto, Ontario, Canada; 7 Department of Medicine, Sunnybrook Health Sciences Centre, Toronto, Ontario, Canada

## Abstract

**Importance:**

Older adults are at greater risk of cognitive decline with various oncologic therapies. Some commonly used therapies for advanced prostate cancer, such as enzalutamide, have been linked to cognitive impairment, but published data are scarce, come from single-group studies, or focus on self-reported cognition.

**Objective:**

To longitudinally examine the association between cognitive function and docetaxel (chemotherapy), abiraterone, enzalutamide, and radium Ra 223 dichloride (radium 223) in older men with metastatic castration-resistant prostate cancer.

**Design, Setting, and Participants:**

A multicenter, prospective, observational cohort study was conducted across 4 academic cancer centers in Ontario, Canada. A consecutive sample of 155 men age 65 years or older with metastatic castration-resistant prostate cancer starting any treatment with docetaxel, abiraterone acetate, enzalutamide, or radium Ra 223 dichloride (radium 223) were enrolled between July 1, 2015, and December 31, 2019.

**Exposures:**

First-line chemotherapy (docetaxel), abiraterone, enzalutamide, or radium 223.

**Main Outcomes and Measures:**

Cognitive function was measured at baseline and end of treatment using the Montreal Cognitive Assessment, the Trail Making Test part A, and the Trail Making Test part B to assess global cognition, attention, and executive function, respectively. Absolute changes in scores over time were analyzed using univariate and multivariable linear regression, and the percentages of individuals with a decline of 1.5 SDs in each domain were calculated.

**Results:**

A total of 155 men starting treatment with docetaxel (n = 51) (mean [SD] age, 73.5 [6.2] years; 34 [66.7%] with some postsecondary education), abiraterone (n = 29) (mean [SD] age, 76.2 [7.2] years; 18 [62.1%] with some postsecondary education), enzalutamide (n = 54) (mean [SD] age, 75.7 [7.4] years; 33 [61.1%] with some postsecondary education), and radium 223 (n = 21) (mean [SD] age, 76.4 [7.2] years; 17 [81.0%] with some postsecondary education) were included. Most patients had stable cognition or slight improvements during treatment. A cognitive decline of 1.5 SDs or more was observed in 0% to 6.5% of patients on each measure of cognitive function (eg, 3 of 46 patients [6.5%; 95% CI, 2.2%-17.5%] in the group receiving chemotherapy [docetaxel] had a decline of 1.5 SDs for Trails A and Trails B). Although patients taking enzalutamide had numerically larger declines than those taking abiraterone, differences were small and clinically unimportant.

**Conclusions and Relevance:**

These findings suggest that most older men do not experience significant cognitive decline in attention, executive function, and global cognition while undergoing treatment for advanced prostate cancer regardless of the treatment used.

## Key Points

**Question:**

Are commonly used treatments for metastatic castration-resistant prostate cancer (mCRPC) associated with a decline in cognitive function in older men?

**Findings:**

This cohort study followed up 198 men aged 65 years or older with mCRPC treated with docetaxel, abiraterone acetate, enzalutamide, or radium Ra 223 dichloride. More than 90% of patients had stable or only slightly reduced cognition (measured using the Trail Making Test part A, the Trail Making Test part B, and the Montreal Cognitive Assessment), with minimal differences among treatments.

**Meaning:**

These findings suggest that most older men do not experience significant cognitive decline while undergoing treatment for mCRPC regardless of the treatment used.

## Teaser

This cohort study examines the association between cognitive function and chemotherapy in older men with metastatic castration-resistant prostate cancer.

## Introduction

Prostate cancer is the most common form of noncutaneous cancer in men.^1^ Prostate cancer predominantly affects older men, with 1 in 5 patients diagnosed at 75 years or older.^2^ For men presenting with advanced disease, androgen deprivation therapy (ADT) is often the first line of treatment.^3^ Unfortunately, most men undergoing ADT will eventually progress to metastatic castration-resistant prostate cancer (mCRPC).^4^ Advanced prostate cancer (including castration-sensitive and castration-resistant disease) is increasingly managed with androgen axis–targeted therapies, such as abiraterone acetate and enzalutamide, docetaxel-based chemotherapy, or radium Ra 223 dichloride (radium 223), all of which have been reported to prolong survival in 1 or more settings.^4^ Choosing among these options and sequencing agents is complex, raising questions about differential adverse effects, such as cognitive decline. Preserving function and cognition are high priorities for older adults with cancer who are considering cancer treatment.^5^

Cognitive function is an important consideration in older adults because increased age is a risk factor for cognitive impairment.^6^ As many as one-third of adults 85 years or older have dementia.^6^ Older patients receiving oncologic therapies are at even greater risk for cognitive decline for multiple reasons, particularly comorbidity and medication effects.^2^ Although numerous studies^7^ have examined the cognitive adverse effects of ADT, few studies^8^ have examined other treatments for advanced prostate cancer.

Previous studies^8,9^ have reported broad associations between chemotherapy and cognitive decline (known as chemo brain), but studies specific to docetaxel and prostate cancer could not be found. Abiraterone and enzalutamide are generally well tolerated, but their effects on cognition may differ. Enzalutamide crosses the blood-brain barrier, carrying an increased risk of seizures, fatigue, and other central nervous system effects.^10,11^ Similarly, abiraterone has been associated with fatigue but does not cross the blood-brain barrier or cause seizures.^10,11^ To our knowledge, only 2 comparative studies^12,13^ examining the association of abiraterone and enzalutamide with objective cognitive function have been performed to date. Neither study reported a clear cognitive advantage of either treatment. However, 2 studies^13,14^ examined self-reported cognitive measures and found that patients treated with enzalutamide reported significantly higher rates of perceived cognitive impairment compared with abiraterone. Although radium 223 is generally well tolerated,^15^ no studies examining its cognitive effects could be identified. Duration of therapy may also influence cognition, but no data in the prostate cancer setting were found. Given how widely these agents are being used in men with advanced prostate cancer, additional studies are warranted.

We conducted a multicenter, prospective cohort study to examine the association between cognition and commonly used treatments (docetaxel, abiraterone, enzalutamide, and radium 223) for advanced prostate cancer in older men. The hypotheses were as follows. Chemotherapy would be associated with the highest rate of cognitive decline based on previous studies^8,9^ in the general cognition and cancer treatment literature. Enzalutamide would be associated with a higher degree of cognitive decline than abiraterone but to a similar or lesser degree than chemotherapy because enzalutamide is known to cross the blood-brain barrier (and cause central nervous system adverse effects that are not seen with abiraterone) but has not been directly linked to cognitive decline in contrast to chemotherapy.^10,11,12,13,14,15,16^

## Methods

### Study Design and Setting

**Figure 1.**
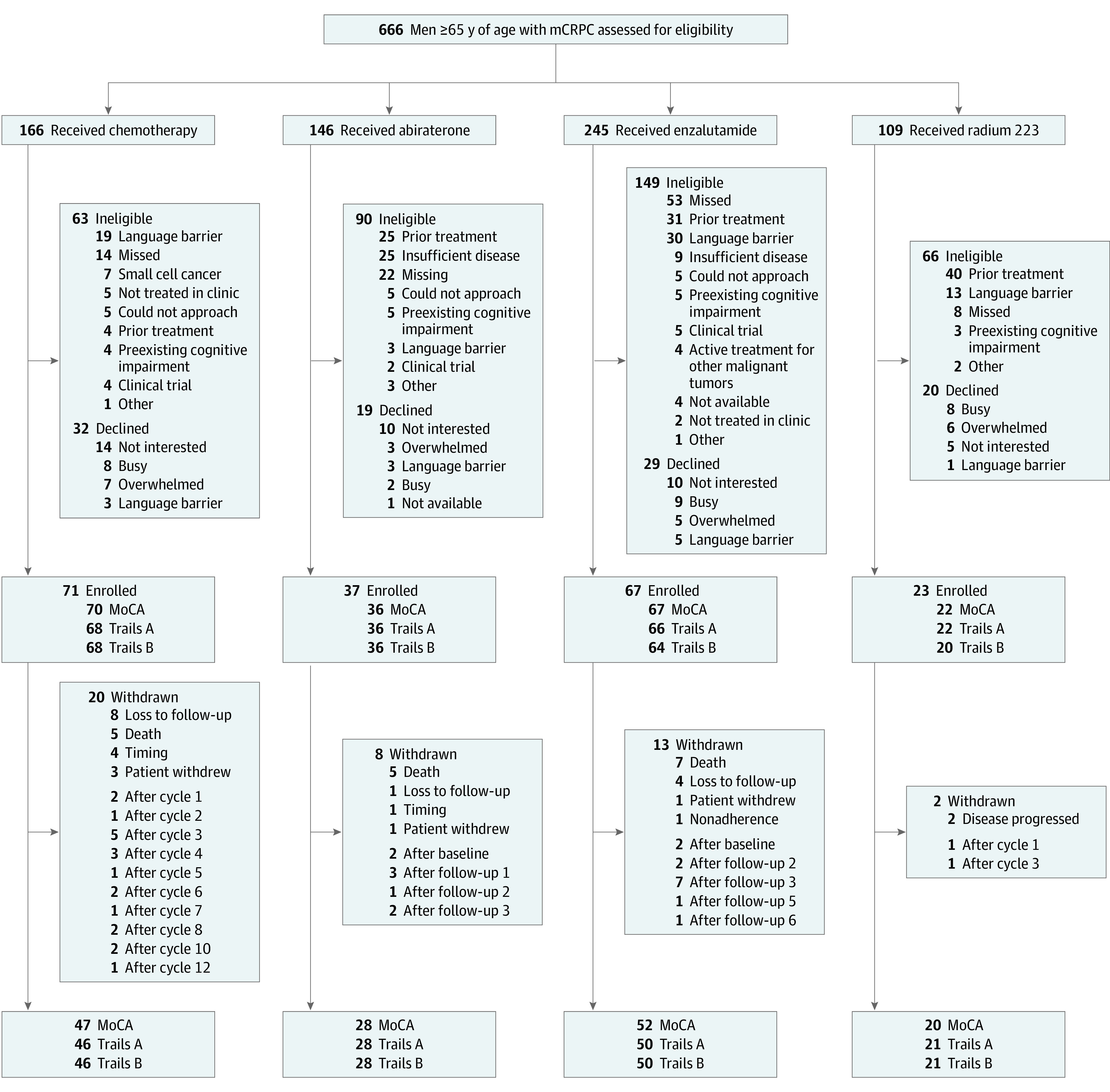
Diagram Illustrating the Flow of Participants Through the Study mCRPC indicates metastatic castration-resistant prostate cancer; MoCA, Montreal Cognitive Assessment; radium 223, radium Ra 223 dichloride; Trails, Trail Making Test.

This prospective, multicenter, observational cohort study (Towards Optimal Prescription of Chemotherapy in Prostate Cancer [TOPCOP1]) enrolled 4 cohorts of men and was completed during 4 years (July 1, 2015, to December 31, 2019). Cognitive function was measured at 2 points: before starting treatment and at the end of treatment (after 6 months for docetaxel and radium 223 and approximately 11 months for abiraterone and enzalutamide). The flow of participants is illustrated in Figure 1. The study involved 4 university-affiliated Canadian centers: the Princess Margaret Cancer Centre (Toronto, Ontario), the Odette Cancer Centre (Toronto, Ontario), the Kingston Health Sciences Centre (Kingston, Ontario), and the Juravinski Cancer Centre (Hamilton, Ontario). This study was approved by the Ontario Cancer Research Ethics Board and at each participating institution, and all patients provided written informed consent. There were a series of safeguards for data containing personal health information per the institutional review board. Data shared among the 4 participating institutions were deidentified. This study followed the Strengthening the Reporting of Observational Studies in Epidemiology (STROBE) reporting guideline.

### Participants

A consecutive sample of participants was recruited. Participants were men diagnosed with mCRPC at least 65 years of age who were about to start their first line of either chemotherapy (docetaxel), abiraterone, enzalutamide, or radium 223 (all commonly used and approved therapies for mCRPC). All participants were receiving ADT with a luteinizing hormone–releasing hormone agent. Participants in the chemotherapy and radium 223 cohorts could have had prior exposure to abiraterone or enzalutamide in the mCRPC setting. Participants were excluded if they were not fluent in English, had a major neuropsychiatric abnormality, or had a life expectancy of fewer than 3 months.

### Materials

Three objective measures of cognition, the Montreal Cognitive Assessment (MoCA), Trail Making Test part A (Trails A), and Trail Making Test part A (Trails B), were administered at baseline and end of study. Our choice of measures was guided by several factors, including feasibility, patient burden, cost, prior use in men with prostate cancer, and recommendations from the International Cognition and Cancer Task Force (ICCTF).^17^

In general, the Trails A task assessed perceptual-motor function by requiring participants to connect a sequence of 25 numbers in order, whereas the Trails B task assessed executive function by requiring participants to connect an alternating sequence of 25 numbers and letters.^18^ Construct validity of the Trail Making Tests has been demonstrated through correlations with the Wechsler Adult Intelligence Scale (Digit Symbol Test for Trails A and Digit Backward Test for Trails B).^19^ Reliability coefficients are generally higher for the Trails B task (often ≥0.65) compared with the Trails A task, but moderate variation has been observed, depending on the patient population.^20^

MoCA was used to assess global cognition by requiring participants to complete a series of brief tests spanning 8 cognitive abilities (short-term memory, visuospatial, executive function, attention, concentration, working memory, language, and orientation).^21^ Concurrent validity between MoCA and the Mini-Mental State Examination has been found (*r* = 0.87).^21^ Test-retest reliability after approximately 1 month has also been excellent (*r* = 0.92).^21^

The Geriatric 8 tool is a practitioner-administered geriatric risk screening tool with a focus on quality of life and functionality.^22^ It incorporates risk factors, such as appetite, weight loss, and mobility. The Vulnerable Elders Survey 13 was used to determine frailty.^23^ This 13-item self-assessment questionnaire measured self-rated health, physical function, and activities of daily living. These 2 measures were used to characterize the underlying frailty and geriatric risk of study participants.

The Instrumental Activities of Daily Living (IADL) questionnaire is 1 of the oldest measurements of daily functioning.^24^ The IADL was used to assess each participant’s ability to perform activities that improve quality of life, such as shopping, preparing food, and handling medications.

### Procedures

Participants were recruited from genitourinary clinics and chemotherapy day units. Baseline cognitive function was assessed^18,19,20,21^ by a trained research assistant. The Geriatric 8, the Vulnerable Elders Survey 13, and the IADL questionnaires were also administered at baseline to assess frailty and functioning.^22,23,24^ Participants were reevaluated at the end of their treatment (after 6 months for docetaxel and radium 223 and approximately 11 months for abiraterone and enzalutamide) with the same cognitive tests. This time was chosen to answer the question of cognitive associations with a given course of treatment.

### Statistical Analysis

Statistical analyses were performed using SAS software, version 9.4 (SAS Institute Inc). Baseline characteristics were described using means for continuous variables and proportions or counts for categorical variables. Between-group comparisons were performed with analysis of covariance or χ^2^ tests as appropriate.

Our primary objective was a within-cohort analysis of change over time. Two analytic approaches were used to explore change over time. First, absolute changes in cognitive scores over time were analyzed per cohort using paired *t* tests. To examine factors associated with change, we used univariate and multivariable linear regression. Age, educational attainment, and treatment cohort were retained in the model based on a prior study^25^ examining cognition in prostate cancer, and other variables with a univariate *P* < .20 were included with no further stepwise selection.^26^ Second, the percentage of individuals with a decrease of 1.5 SDs or more in 2 or more cognitive tests was calculated, as per the ICCTF guidelines.^16^ We also reported the percentage of individuals declining by at least 1.5 SDs on each test along with the 95% CIs calculated using the Wilson interval method. A 2-sided *P* < .05 was considered statistically significant. No adjustment was made for multiple comparisons.^27^

Cognitive outcomes were a prespecified secondary outcome in TOPCOP1. However, no formal sample size calculation was performed for secondary outcomes. Instead, we aimed to recruit 75 patients starting chemotherapy. and we accrued men to all other cohorts until 75 men starting chemotherapy were recruited. To assess the impact of any missing data and possible attrition bias, we recorded reasons for premature study discontinuation and examined baseline differences between participants who did and did not provide outcome data at the end of treatment.

## Results

### Baseline Characteristics

A total of 155 men starting treatment with docetaxel (n = 51) (mean [SD] age, 73.5 [6.2] years; 34 [66.7%] with at least some postsecondary education), abiraterone (n = 29) (mean [SD] age, 76.2 [7.2] years; 17 [81.0%] with at least some postsecondary education), enzalutamide (n = 54) (mean [SD] age, 75.7 [7.4] years; 33 [61.1%] with at least some postsecondary education), and radium 223 (n = 21) (mean [SD] age, 76.4 [7.2] years; 16 [76.2%] with at least some postsecondary education) were included. Of the 666 patients approached for the study, 368 were ineligible (primarily because of prior treatment, language barriers, and administrative reasons) (Figure 1), and 100 declined participation. Of the 198 consecutively enrolled patients, 43 participants were withdrawn from the study primarily because of disease progression or death, and 157 participants completed the study (completion rate, 79%). The recruitment, enrollment, and withdrawal of participants is illustrated in Figure 1. Some participants did not complete cognitive testing at the final visit, most often because of disease progression or study withdrawal (Figure 1).

Baseline cognitive function was similar between the cohorts for all 3 cognitive measures (Table 1). Baseline characteristics among those who did and did not complete the study were similar in terms of age, educational attainment, and 2 of 3 cognitive measures, whereas participants who did not complete the study had worse Trails B scores at baseline.

**Table 1.** Baseline Demographic and Clinical Characteristics

Characteristic	Patients by treatment, No. (%)				*P* value
	Chemotherapy (docetaxel) (n = 71)	Abiraterone acetate (n = 37)	Enzalutamide (n = 67)	Radium Ra 223 dichloride (n = 23)	
**Sociodemographic factors**					
Age, mean (SD) [range], y	73.5 (6.2) [65-90]	76.2 (7.2) [65-93]	75.7 (7.4) [65-97]	76.4 (7.2) [65-89]	.14
Educational attainment					
Graduate education	20 (28.2)	10 (27.0)	11 (16.4)	4 (17.4)	.19
Undergraduate university or college	24 (33.8)	12 (32.4)	18 (26.9)	11 (47.8)	
Some university or college	6 (8.5)	2 (5.4)	12 (17.9)	3 (13.0)	
Completed high school	9 (12.7)	9 (24.3)	13 (19.4)	2 (8.7)	
Some high school	3 (4.2)	3 (8.1)	6 (8.9)	3 (13.0)	
Elementary or middle school	9 (12.7)	1 (2.7)	7 (10.5)	0 (0)	
Working full time or part time	14 (19.7)	9 (24.3)	11 (16.4)	1 (4.3)	.24
Living alone	11 (15.5)	3 (8.1)	11 (16.4)	3 (13)	.68
**Clinical variables**					
Comorbidities					
Anemia	65 (91.6)	23 (62.2)	49 (73.1)	16 (69.6)	.002
Neurologic	8 (11.3)	4 (10.8)	8 (11.9)	3 (13.0)	.99
Cardiovascular	46 (64.8)	25 (67.6)	49 (73.1)	13 (56.5)	.48
Musculoskeletal	35 (49.3)	22 (59.5)	30 (44.8)	15 (65.2)	.26
Diabetes	16 (22.5)	4 (10.8)	22 (32.8)	5 (21.8)	.09
Medication burden, median count (IQR)	5 (3-7)	4 (2-6)	4 (2-7)	5 (3-8)	.27
ECOG PS 0-1	66 (92.9)	35 (94.6)	64 (95.5)	22 (95.7)	.84
IADL, fully independent	31 (43.7)	24 (64.9)	42 (62.7)	12 (52.2)	.08
VES-13 score >3	33 (46.5)	8 (21.6)	22 (32.8)	8 (34.8)	.07
G8 score <14	52 (73.2)	17 (45.9)	35 (52.2)	9 (39.1)	.02
**Oncologic variables**					
Time since mCRPC diagnosis, median (IQR), mo	19.7 (7.7-37.2)	2.6 (1.1-5.6)	2.1 (1.3-6.8)	1.4 (3.5-3.6)	.71
ADT duration, median (IQR), mo^a^	55.8 (33.7-103.9)	50.5 (27.9-88.4)	50.0 (26.3-110.2)	56.2 (27.9-110.9)	.62
Grade group at diagnosis					
1	5 (7.0)	3 (8.1)	11 (16.4)	0	.04
2	9 (12.7)	2 (5.4)	10 (14.9)	9 (39.1)	
3	16 (22.5)	7 (18.9)	15 (22.4)	5 (21.7)	
4	8 (11.3)	4 (10.8)	6 (8.9)	2 (8.7)	
5	23 (32.4)	19 (51.4)	18 (26.9)	4 (17.4)	
Not available	10 (14.1)	2 (5.4)	7 (10.5)	3 (13.0)	
Sites of metastatic involvement					
Bone	58 (81.7)	26 (70.3)	48 (71.6)	23 (100)	.02
Lymph node	27 (38.0)	16 (43.2)	33 (49.3)	4 (17.4)	.06
Visceral	12 (16.9)	0	9 (13.4)	1 (4.4)	.02
LDH, median (IQR), IU/L^b^	282 (215-338)	216 (175-259)	207 (182-245)	233 (200-257)	.001
ALP, median (IQR), IU/L	113 (80-241)	99 (76-139)	88 (72-115)	105 (65-189)	.03
PSA, median (IQR), ng/mL	72.2 (29.4-167.6)	15.1 (5.0-33.0)	19.6 (5.7-40.3)	43.9 (8.8-123.7)	.02
Total testosterone, median (IQR), ng/dL	17.3 (5.8-17.3))	14.4 (11.5-20.2)	14.4 (11.5-20.2)	11.5 (11.5-23.1)	.46
Treatment duration of mCRPC treatment, median (IQR), mo	5.2 (3.2-7.5)	11.7 (8.1-15.6)	11.2 (7.4-22.8)	5.7 (4.6-6.1)	<.001
Standard dose^c^	46 (64.8)	36 (100)	64 (95.5)	23 (100)	<.001
Cognitive assessments^d^					
Trails A score, mean (SD) [range], s	51.9 (20.9) [20-90]	51.2 (17.5) [24-90]	54.0 (20.2) [23-90]	52.0 (19.8) [28-90]	.88
Trails B score, mean (SD) [range], s	140.8 (72.8) [58-300]	126.3 (54.7) [61-300]	140.8 (72.8) [54-300]	130.4 (75.9) [49-300]	.51
Total MoCA score, mean (SD) [range]	23.9 (3.7) [13-30]	25.3 (2.8) [18-30]	24.0 (4.2) [13-30]	25.8 (3.7) [17-30]	.08

Abbreviations: ADT, androgen deprivation therapy; ALP, alkaline phosphatase; ECOG PS, Eastern Cooperative Oncology Group Performance Status; G8, Geriatric 8; IADL, Instrumental Activities of Daily Living; IQR, interquartile range; LDH, lactate dehydrogenase; mCRPC, metastatic castration-resistant prostate cancer; MoCA, Montreal Cognitive Assessment; PSA, prostate-specific antigen; Trails, Trail Making Test; VES-13, Vulnerable Elders Survey 13.

SI conversion factors: To convert ALP to microkatals per liter, multiply by 0.0167; LDH to microkatals per liter, multiply by 0.0167; PSA to micrograms per liter, multiply by 1; and total testosterone to nanomoles per liter, multiply by 0.0347.

^a^ Duration from first start of ADT to first start of abiraterone, enzalutamide, chemotherapy, or radium Ra 223 dichloride therapy.

^b^ Lactate dehydrogenase was not measured in 1 center.

^c^ Standard dose was 1000 mg of abiraterone acetate and 10 mg of prednisone daily, 160 mg of enzalutamide daily, 75 mg/m^2^ of docetaxel, or 55 kBq/kg of radium Ra 223 dichloride.

^d^ Higher scores represent worse cognitive performance on the Trails A and B tests but better performance on MoCA.

### Mean Change in Cognitive Scores

Each cohort had similar scores at baseline and final visit (Table 2). In general, cognitive scores remained stable or improved slightly on all 3 measures in all 4 cohorts, but none of the change scores were statistically different from 0 (Table 2).

**Table 2.** Cognitive Scores at Baseline and Final Visit for Each Treatment Cohort^a^

Cognitive measure	Chemotherapy (docetaxel) (n = 51)	Abiraterone acetate (n = 29)	Enzalutamide (n = 54)	Radium Ra 223 dichloride (n = 21)
Trails A, s	(n = 46)	(n = 28)	(n = 50)	(n = 21)
Baseline score, mean (SD)	54.4 (21.9)	45.1 (11.3)	51.3 (19.9)	53.2 (20.4)
Final visit score, mean (SD)	50.8 (19.6)	47.5 (13.3)	50.7 (20.4)	50.0 (22.3)
Change (95% CI)	–3.6 (–8.4 to 1.3)	2.4 (–2.1 to 7.0)	–0.6 (–3.7 to 2.4)	–3.2 (–7.4 to 1.0)
*P* value^b^	.14	.28	.68	.13
Trails B, s	(n = 46)	(n = 28)	(n = 50)	(n = 21)
Baseline score, mean (SD)	142.1 (75.9)	112.6 (44.9)	133.1 (65.9)	133.8 (78.5)
Final visit score, mean (SD)	129.8 (70.7)	123.9 (55.2)	135.7 (69.9)	125.6 (86.1)
Change (95% CI)	–12.3 (–30.0 to 5.4)	11.3 (–11.4 to 33.9)	2.6 (–8.9 to 14.1)	–8.2 (–36.0 to 19.6)
*P* value^b^	.17	.32	.65	.54
Total MoCA	(n = 47)	(n = 28)	(n = 52)	(n = 20)
Baseline score, mean (SD)	24.0 (3.6)	25.3 (2.8)	24.8 (4.1)	25.6 (3.7)
Final visit score, mean (SD)	24.5 (4.5)	25.5 (2.6)	24.5 (3.3)	24.1 (4.2)
Change (95% CI)	0.5 (–0.4 to 1.4)	0.2 (–0.8 to 1.1)	–0.3 (–1.1 to 0.5)	–1.5 (–2.9 to 0.0)
*P* value^b^	.29	.70	.43	.06
Individuals with decline of at least 1.5 SDs for each treatment cohort, No./total No. (%) [95% CI]^c^				
Trails A^d^	3/46 (6.5) [2.2 to 17.5]	0 (0) [0 to 12.1]	0 (0) [0 to 7.1]	0 (0) [0 to 15.4]
Trails B^e^	3/46 (6.5) [2.2 to 17.5]	0 (0) [0 to 12.1]	0 (0) [0 to 7.1]	1/21 (4.8) [0.9 to 22.7]
Total MoCA^f^	2/47 (4.3) [1.2 to 14.3]	0 (0) [0 to 12.1]	1/52 (1.9) [0.3 to 10.5]	1/20 (5.0) [0.9 to 23.6]

Abbreviations: MoCA, Montreal Cognitive Assessment; Trails, Trail Making Test.

^a^ Lower scores on Trails A and B and higher scores on MoCA are indicative of improvement.

^b^ *P* values were obtained from paired *t* tests.

^c^ The 95% CIs were generated using the Wilson interval method.

^d^ *P* = .08.

^e^ *P* = .48.

^f^ *P* = .89.

### Decline of at Least 1.5 SDs

**Figure 2.**
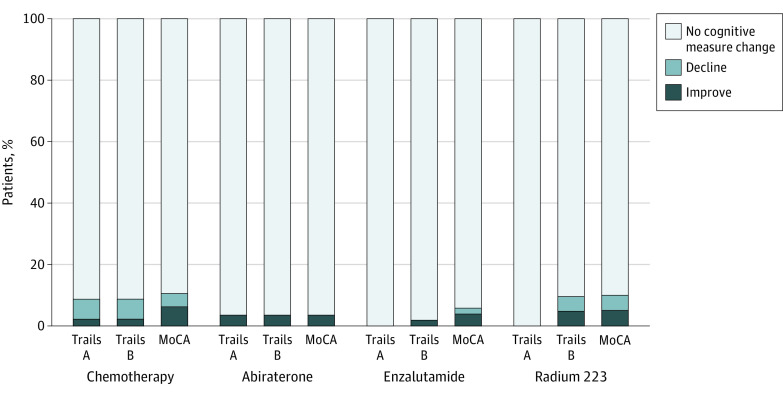
Proportion of Patients With a Change of at Least 1.5 SDs on Each Cognitive Assessment MoCA indicates Montreal Cognitive Assessment; Trails, Trail Making Test.

No patient experienced a clinically significant cognitive decline as defined by the ICCTF (decline of at least 1.5 SDs on ≥2 measures).^16^ A mean of 0% to 6.5% of patients had declined on each measure of cognitive function by at least 1.5 SDs. For example, eg, 3 of 46 patients (6.5%; 95% CI, 2.2%-17.5%) in the group receiving chemotherapy (docetaxel) had a decline of 1.5 SDs for Trails A and Trails B. In contrast, 90% to 100% of patients in each treatment group had no clinically important change from baseline. For example, no patients in the abiraterone group (0%; 95% CI, 0%-12.1%) showed a decline of 1.5 SDs on the Trails A, Trails B, or MoCA (Table 2, Figure 2). Cohorts treated with chemotherapy and radium 223 had a greater number of participants with worsening scores by at least 1.5 SDs, but these differences were not statistically significant (Table 2, Figure 2).

### Factors Associated With Cognitive Decline

In the univariate linear regression, increasing age was associated with a greater decline in Trails A score (β = 8.84 for age ≥85 years compared with age 65-74 years, *P* = .03) but not the other 2 cognitive tests (β = 6.06 for age ≥85 years compared with age 65-74 years for Trails B; β = 0.35 for age ≥85 years compared with age 65-74 years for total MoCA). Educational attainment (at least some postsecondary education vs high school or less) (β = −0.79 for Trails A, β = −7.47 for Trails B, and β = −0.04 for MoCA), frailty (β = 2.49 for Trails A, β = −7.47 for Trails B, and β = −0.36 for MoCA), IADL status (β = 0.45 for Trails A, β = 7.19 for Trails B, and β = −0.07 for MoCA), and performance status (β = 2.42 for Trails A, β = 15.62 for Trails B, and β = −1.48 for MoCA) were not associated with a cognitive decline on any test in the univariate models. Other than baseline test score (β = −0.27 for Trails A, β = −0.32 for Trails B, and β = −0.31 for MoCA), no other variable, including treatment group, was significantly associated with cognitive decline in the multivariable models (eTable in the Supplement). Dose of chemotherapy was not associated with cognitive decline in that treatment group.

## Discussion

This cohort study examined objective cognitive function over time in older men starting 1 of 4 common treatments for advanced prostate cancer. Patients had no decline in attention, executive function, or global cognition in each treatment group, with no appreciable difference among treatments. Another analytic approach recommended by the ICCTF was also used. These analyses revealed that most patients in each treatment group had stable cognitive function between the start and end of treatment, and no patient declined by at least 1.5 SDs on 2 or more cognitive measures. The study did not identify any consistent association between cognitive decline and advanced prostate cancer.

The results of this study partially supported the study hypotheses. Although the chemotherapy group had the largest proportion of patients with a decline in cognition, these numbers were still low and neither statistically different from the other 3 treatments nor clinically meaningful based on the ICCTF criteria. Cognitive declines were similar in patients treated with enzalutamide and abiraterone. Interestingly, radium 223 was observed to be associated with minor cognitive declines on 1 cognitive measure. This decline was not statistically significant, and given the small sample size, these findings are exploratory and further studies are warranted. Overall, the results did not indicate that any of the 4 treatments for advanced prostate cancer were associated with an important decline in cognitive function using 2 complementary analytic approaches.

Research into the effects of cancer treatment on cognition is increasing, although results have varied depending on study methods. Subjective measures of cognitive function (ie, self-reported outcomes) suggest a large proportion of people undergoing systemic therapy experience cognitive decline; however, studies using objective neuropsychological measures have been mixed.^9^ This discrepancy could be explained by studies^28,29^ suggesting that self-reported cognitive function correlates more with psychological measures of distress (eg, depression and anxiety) than with objective cognitive function. Moreover, most studies^9^ on cancer treatment–related cognitive decline have used samples of high-functioning young women with breast cancer undergoing chemotherapy or hormonal therapy. The generalizability of these findings to older men with prostate cancer is unclear.

Three prior studies^12,13,30^ have examined objective cognitive function with at least 1 agent in the mCRPC setting.^8^ In a single-arm study, Gotto et al^30^ reported a small change in MoCA in men taking abiraterone (mean difference, <1 point) after 12, 24, 48, and 72 weeks of treatment. A study by Khalaf et al^12^ examined the proportion of men who developed an abnormal MoCA score (<26 of 30) during treatment with abiraterone or enzalutamide and found no difference (47% vs 54%, *P* = .40). Finally, Shore et al^13^ used a computerized cognitive battery (Cogstate) to examine the proportion of men with mCRPC who declined using a reliable change index approach. They reported 6% of men taking enzalutamide vs 2% taking abiraterone declined (*P* value not reported). The results of the current study comparing cognitive changes over time with abiraterone and enzalutamide are similar to the studies by Khalaf et al^12^ and Shore et al,^13^ which did not find significant differences between the 2 agents. The magnitude of change in MoCA scores observed in the current study is also similar to those observed by Gotto et al^30^ and Shore et al.^13^ Although 2 studies^13,14^ using subjective measures of cognition found that patients treated with enzalutamide reported significantly greater cognitive-related symptoms (eg, fatigue, memory impairment, and confusion) compared with abiraterone, the clinical significance of such symptoms is unclear, and the current study did not assess cognitive symptoms. The current study could not identify any prior studies examining the cognitive effects of docetaxel or radium 223 in this population with which to compare our results.

### Strengths and Limitations

This study has several strengths. It is the first, to our knowledge, to explore the association between cognition and docetaxel and radium 223. It is also the first, to our knowledge, to include docetaxel, abiraterone, enzalutamide, and radium 223 using objective neuropsychological measures. This study focused on older adults, who have historically been excluded in clinical oncology research, despite being at a higher risk of cognitive decline.^6,17^ Thus, this research adds substantially to the limited published studies describing the cognitive changes of older men with advanced prostate cancer, which is significant because older adults with cancer are less willing than younger adults to accept adverse effects (particularly cognitive or functional decline) in return for gains in survival.^31,32^

This study also has limitations that should be kept in mind when interpreting the findings. First, although a total of 198 participants were enrolled in the study, each cohort had a modest sample size, particularly the radium 223 group. The lack of an untreated control group also made it difficult to know whether changes in cognitive function were specifically attributable to treatment. However, on the basis of the 95% CIs, most men would be unlikely to demonstrate significant cognitive declines as defined by the ICCTF in larger studies because the upper confidence limit for such a decline is less than 20% of men for each treatment (Table 2). In addition, the 95% CI around the absolute differences in each outcome also excludes major declines. For example, the minimum clinically important difference are approximately 10 seconds for Trails A, 35 seconds for Trails B, and 2 points for MoCA. The CIs for each of these outcomes for all 4 treatments were smaller than the minimum clinically important difference (Table 2), suggesting that few men would have a noticeable decline in any of these cognitive measures. Of importance, however, small between-group differences in cognition cannot be ruled out given the study design and sample size, and larger studies would be required.

Whether the current study’s findings can be extrapolated to other agents, such as apalutamide, darolutamide, or cabazitaxel, is unclear. In addition, the absence of randomization may have led to selection bias and imbalances between groups. Because practitioners ultimately determined the allocation of treatments, access to treatment and which treatments were deemed appropriate may have varied among patients. Although age, educational attainment, and cognitive scores were similar among the cohorts at baseline, these results are best viewed as providing estimates of within-cohort change that can help practitioners discuss potential cognitive adverse effects of an agent that they are considering for their patient. Survivorship bias may also be in effect because patients who had died before the end-of-study visit could not be included and may have systematically differed from those who survived. Nevertheless, baseline characteristics among those who did and did not provide end-of-study data were mostly similar. Although the time between assessments differed between the oral agents and the intravenous therapies by a mean of 6 months, larger cognitive declines were not detected with abiraterone or enzalutamide compared with docetaxel or radium 223, and the results answer the clinically relevant question of change in cognition during treatment, which is arguably the most relevant to patients. However, it would be interesting to study cognitive changes over time among long-term users of androgen axis–targeted agents, some of whom continued to receive treatment for several years. In the current study, a limited selection of cognitive measures was used. However, the study contributes important data in this field, particularly for docetaxel and radium 223, for which there are no published data. Although all men were undergoing conventional ADT, this was unlikely to influence the study findings given that the assessments were performed at the beginning and end of treatment. However, because all men had mCRPC, it is unclear whether the findings are equally applicable to earlier stages of advanced prostate cancer. Furthermore, because no self-reported cognitive measures were included in the study, the data do not allow us to explain previously reported differences between enzalutamide and abiraterone on self-reported cognitive function.

## Conclusions

Using 3 common, objective measures of cognitive function, this cohort study was unable to detect significant cognitive decline in any of the 4 treatments for advanced prostate cancer. However, given the limitations of this study, future trials using a more thorough neuropsychological assessment, self-reported measures, and a larger sample with multiple times are warranted.

## Notes

Supplement. **eTable.** Linear Regression of Factors Associated With Change for Each Cognitive Measure

## References

[1] Siegel RL, Miller KD, Jemal A. Cancer statistics, 2020. CA Cancer J Clin. 2020;70(1):7-30. doi:10.3322/caac.21590 31912902

[2] Boukovala M, Spetsieris N, Efstathiou E. Systemic treatment of prostate cancer in elderly patients: current role and safety considerations of androgen-targeting strategies. Drugs Aging. 2019;36(8):701-717. doi:10.1007/s40266-019-00677-6 31172421

[3] Sharifi N, Gulley JL, Dahut WL. Androgen deprivation therapy for prostate cancer. JAMA. 2005;294(2):238-244. doi:10.1001/jama.294.2.238 16014598

[4] Nuhn P, De Bono JS, Fizazi K, . Update on systemic prostate cancer therapies: management of metastatic castration-resistant prostate cancer in the era of precision oncology. Eur Urol. 2019;75(1):88-99. doi:10.1016/j.eururo.2018.03.028 29673712

[5] Rocque GB, Williams GR. Bridging the data-free zone: decision making for older adults with cancer. J Clin Oncol. 2019;37(36):3469-3471. doi:10.1200/JCO.19.02588 31675251 PMC7194450

[6] Dementia in Canada, Including Alzheimer’s Disease. Public Health Agency of Canada. Accessed May 27, 2021. https://www.canada.ca/content/dam/phac-aspc/documents/services/publications/diseases-conditions/dementia-highlights-canadian-chronic-disease-surveillance/dementia-highlights-canadian-chronic-disease-surveillance.pdf

[7] Sun M, Cole AP, Hanna N, . Cognitive impairment in men with prostate cancer treated with androgen deprivation therapy: a systematic review and meta-analysis. J Urol. 2018;199(6):1417-1425. doi:10.1016/j.juro.2017.11.136 29410294

[8] Batra A, Marchioni M, Hashmi AZ, . Cognition and depression effects of androgen receptor axis-targeted drugs in men with prostate cancer: a systematic review. J Geriatr Oncol. Published online November 22, 2020;S1879-4068(20)30494-X. doi:10.1016/j.jgo.2020.11.002 33234494

[9] Tschernichovsky R, Philp L, Goodman A. Chemotherapy-induced cognitive decline: moving from the mechanistic debate towards prevention and treatment—a clinical review. J Cancer Ther. 2019;10(12):985-1012. doi:10.4236/jct.2019.1012084

[10] Ryan C, Wefel JS, Morgans AK. A review of prostate cancer treatment impact on the CNS and cognitive function. Prostate Cancer Prostatic Dis. 2020;23(2):207-219. doi:10.1038/s41391-019-0195-5 31844181 PMC7237350

[11] Moreira RB, Debiasi M, Francini E, . Differential side effects profile in patients with mCRPC treated with abiraterone or enzalutamide: a meta-analysis of randomized controlled trials. Oncotarget. 2017;8(48):84572-84578. doi:10.18632/oncotarget.20028 29137449 PMC5663621

[12] Khalaf DJ, Sunderland K, Eigl BJ, . Health-related quality of life for abiraterone plus prednisone versus enzalutamide in patients with metastatic castration-resistant prostate cancer: results from a phase II randomized trial. Eur Urol. 2019;75(6):940-947. doi:10.1016/j.eururo.2018.12.015 30591354

[13] Shore ND, Saltzstein D, Sieber P, . Results of a Real-world Study of Enzalutamide and Abiraterone Acetate With Prednisone Tolerability (REAAcT). *Clin Genitourin Cancer*. 2019;17(6):457-463, e6. doi:10.1016/j.clgc.2019.07.017 31473120

[14] Thiery-Vuillemin A, Poulsen MH, Lagneau E, ; AQUARiUS Investigators. Impact of abiraterone acetate plus prednisone or enzalutamide on patient-reported outcomes in patients with metastatic castration-resistant prostate cancer: final 12-mo analysis from the observational AQUARiUS Study. Eur Urol. 2020;77(3):380-387. doi:10.1016/j.eururo.2019.09.019 31594705

[15] Harrison MR, Wong TZ, Armstrong AJ, George DJ. Radium-223 chloride: a potential new treatment for castration-resistant prostate cancer patients with metastatic bone disease. Cancer Manag Res. 2013;5:1-14. doi:10.2147/CMAR.S25537 23326203 PMC3544343

[16] Wefel JS, Vardy J, Ahles T, Schagen SB. International Cognition and Cancer Task Force recommendations to harmonise studies of cognitive function in patients with cancer. Lancet Oncol. 2011;12(7):703-708. doi:10.1016/S1470-2045(10)70294-1 21354373

[17] Hurria A, Dale W, Mooney M, ; Cancer and Aging Research Group. Designing therapeutic clinical trials for older and frail adults with cancer: U13 conference recommendations. J Clin Oncol. 2014;32(24):2587-2594. doi:10.1200/JCO.2013.55.0418 25071116 PMC4129504

[18] Spreen O, Strauss E. A Compendium of Neuropsychological Tests. Oxford University Press; 1998.

[19] Sánchez-Cubillo I, Periáñez JA, Adrover-Roig D, . Construct validity of the Trail Making Test: role of task-switching, working memory, inhibition/interference control, and visuomotor abilities. J Int Neuropsychol Soc. 2009;15(3):438-450. doi:10.1017/S1355617709090626 19402930

[20] Lezak MD, Howieson DB, Bigler ED, Tranel D. Neuropsychological Assessment. 5th ed. Oxford University Press; 2012.

[21] Nasreddine ZS, Phillips NA, Bédirian V, . The Montreal Cognitive Assessment, MoCA: a brief screening tool for mild cognitive impairment. J Am Geriatr Soc. 2005;53(4):695-699. doi:10.1111/j.1532-5415.2005.53221.x 15817019

[22] Bellera CA, Rainfray M, Mathoulin-Pélissier S, . Screening older cancer patients: first evaluation of the G-8 geriatric screening tool. Ann Oncol. 2012;23(8):2166-2172. doi:10.1093/annonc/mdr587 22250183

[23] Saliba D, Elliott M, Rubenstein LZ, . The Vulnerable Elders Survey: a tool for identifying vulnerable older people in the community. J Am Geriatr Soc. 2001;49(12):1691-1699. doi:10.1046/j.1532-5415.2001.49281.x 11844005

[24] Lawton MP, Brody EM. Assessment of older people: self-maintaining and instrumental activities of daily living. Gerontologist. 1969;9(3):179-186. doi:10.1093/geront/9.3_Part_1.179 5349366

[25] Alibhai SMH, Breunis H, Timilshina N, . Impact of androgen-deprivation therapy on cognitive function in men with nonmetastatic prostate cancer. J Clin Oncol. 2010;28(34):5030-5037. doi:10.1200/JCO.2010.30.8742 21041708

[26] Harrell FE, Jr. Multivariable modeling strategies. In: Harrell FE Jr, ed. Regression Modeling Strategies: With Applications to Linear Models, Logistic Regression, and Survival Analysis. Springer-Verlag; 2001:53-86. Springer Series in Statistics. doi:10.1007/978-1-4757-3462-1_4

[27] Rothman KJ. No adjustments are needed for multiple comparisons. Epidemiology. 1990;1(1):43-46. doi:10.1097/00001648-199001000-00010 2081237

[28] Zlatar ZZ, Moore RC, Palmer BW, Thompson WK, Jeste DV. Cognitive complaints correlate with depression rather than concurrent objective cognitive impairment in the successful aging evaluation baseline sample. J Geriatr Psychiatry Neurol. 2014;27(3):181-187. doi:10.1177/0891988714524628 24614203 PMC4255945

[29] Savard J, Ganz PA. Subjective or objective measures of cognitive functioning—what’s more important? JAMA Oncol. 2016;2(10):1263-1264. doi:10.1001/jamaoncol.2016.2047 27441735

[30] Gotto G, Drachenberg DE, Chin J, . Real-world evidence in patient-reported outcomes (PROs) of metastatic castrate-resistant prostate cancer (mCRPC) patients treated with abiraterone acetate + prednisone (AA+P) across Canada: final results of COSMiC. Can Urol Assoc J. 2020;14(12):E616-E620. doi:10.5489/cuaj.6388 32569568 PMC7704085

[31] Yellen SB, Cella DF, Leslie WT. Age and clinical decision making in oncology patients. J Natl Cancer Inst. 1994;86(23):1766-1770. doi:10.1093/jnci/86.23.1766 7966414

[32] Uemura H, Matsubara N, Kimura G, . Patient preferences for treatment of castration-resistant prostate cancer in Japan: a discrete-choice experiment. BMC Urol. 2016;16(1):63. doi:10.1186/s12894-016-0182-2 27814714 PMC5095997

