# Error in Key Points

**DOI:** 10.1001/jamanetworkopen.2021.24224

**Published:** 2021-07-29

In the Original Investigation titled “Association of Chemotherapy, Enzalutamide, Abiraterone, and Radium 223 With Cognitive Function in Older Men With Metastatic Castration-Resistant Prostate Cancer,”^1^ published July 2, 2021, the number of men studied was given incorrectly in the Key Points section; it now reads 155. The article was corrected on July 29, 2021.
