# Supplementary material for: Association of Chemotherapy, Enzalutamide, Abiraterone, and Radium 223 With Cognitive Function in Older Men With Metastatic Castration-Resistant Prostate Cancer
Source: JAMA Netw Open. 2021 Jul 2;4(7):e2114694. doi: 10.1001/jamanetworkopen.2021.14694 (PMC8254132; doi:10.1001/jamanetworkopen.2021.14694)
Supplement: Supplement. — eTable. Linear Regression of Factors Associated With Change for Each Cognitive Measure [file jamanetwopen-e2114694-s001.pdf]

Supplementary Online Content

Alibhai SMH, Breunis H, Feng G, et al. Association of chemotherapy, enzalutamide, abiraterone, and radium 223 with cognitive function in older men with metastatic castration-resistant prostate cancer. *JAMA Netw Open*. 2021;4(7):e2114694. doi:10.1001/jamanetworkopen.2021.14694

eTable. Linear Regression of Factors Associated With Change for Each Cognitive Measure

This supplementary material has been provided by the authors to give readers additional information about their work.

© 2021 Alibhai SMH et al. *JAMA Network Open.*

eTable. Linear Regression of Factors Associated With Change for Each Cognitive Measure

	Univariate Model		Multivariate Model	
**Variable**	**β (SE)**	**p-value**	**β (SE)**	**p-value**
Change in Trails A, s				
**Cohort**				
Enzalutamide	Reference		Reference	
Abiraterone	3.04 (3.2)	0.13	0.87 (2.8)	0.75
Chemotherapy	-2.96 (2.6)	0.26	-2.21 (2.6)	0.36
Radium-223	-2.57 (3.3)	0.44	-3.38 (3.2)	0.28
**Age**, per year	0.2 (0.16)	0.21		
**Age Category**				
65-74	Reference		Reference	
75-84	-1.18 (2.23)	0.59	1.68 (2.20)	0.44
85+	**8.84 (4.08)**	**0.032**	**9.54 (4.32)**	**0.029**
**Education**, at least some post secondary vs high school or less	-0.79 (2.26)	0.72	-2.45 (2.07)	0.24
**VES-13**, dichotomous	2.49 (2.3)	0.28	-0.27 (0.05)	0.16
**G8**, dichotomous	1.27 (2.04)	0.53		
**ECOG**, 0-1 vs higher	2.42 (5.38)	0.65		
**IADL**, independent vs dependent<br>**Trails A score at baseline**	0.45 (2.22)<br>**-0.23 (0.06)**	0.84<br>**<0.001**	**-0.27 (0.06)**	**<0.001**
Change in Trails B, s				
**Cohort**				
Enzalutamide	Reference		Reference	
Abiraterone	8.68 (7.8)	0.49	-0.22 (12.08)	0.98
Chemotherapy	-14.92 (10.9)	0.18	-12.85 (10.29)	0.21
Radium-223	-10.79 (13.9)	0.44	-9.76 (13.47)	0.47
**Age**, per year	-0.06 (0.7)	0.92		
**Age Category**				
65-74	Reference		Reference	
75-84	1.56 (9.56)	0.87	9.80 (9.28)	0.29
85+	6.06 (17.45)	0.73	5.73 (17.01)	0.74
**Education**#	-5.02 (9.49)	0.59	-14.73 (8.85)	0.098
**VES-13**, dichotomous	-7.47 (9.75)	0.44		
**G8**, dichotomous	-1.63 (8.55)	0.85		
**ECOG**, 0-1 vs higher	15.62 (22.52)	0.49		
**IADL**, independent vs dependent<br>**Trails B score at baseline**	7.19 (9.28)<br>**-0.29 (0.06)**	0.44<br>**<0.001**	**-0.32 (0.06)**	**<0.001**
Change in Total MoCA, (score 0-30, MCID = 2 points)				
**Cohort**				
Enzalutamide	Reference		Reference	
Abiraterone	0.48 (0.67)	0.47	0.55 (0.65)	0.40

© 2021 Alibhai SMH et al. *JAMA Network Open.*

Chemotherapy	0.78 (0.18)	0.17	0.66 (0.55)	0.23
Radium-223	-1.14 (0.13)	0.13	-0.77 (0.73)	0.29
**Age**, per year	-0.02 (0.04)	0.67		
**Age Category**				
65-74	Reference		Reference	
75-84	-0.36 (0.51)	0.48	-0.96 (0.49)	0.054
85+	0.35 (0.9)	0.7	0.65 (0.94)	0.49
**Education**[#]	-0.04 (0.5)	0.94	0.01 (0.46)	0.98
**VES-13**, dichotomous	-0.36 (0.52)	0.49	-0.19 (0.10)	0.070
**G8**, dichotomous	-0.15 (0.46)	0.73		
**ECOG**, 0-1 vs higher	-1.48 (1.11)	0.21		
**IADL**, independent vs dependent	-0.07 (0.49)	0.88		
**Total MoCA score at baseline**	**-0.28 (0.06)**	**<0.001**	**-0.31 (0.07)**	**<0.001**

Note. VES-13 = Vulnerable Elders-13 Survey; G8 = Geriatric 8; ECOG PS = Eastern Cooperative Oncology Group Performance Status; IADL = Instrumental Activities of Daily Living; MoCA = Montreal Cognitive Assessment. Multivariable model forced in age, education, and cohort; other variables included if p<0.10.

An increase in Trails A and B scores and a decrease in MoCA scores represents worsening performance.

© 2021 Alibhai SMH et al. *JAMA Network Open.*
